# Insomnia, Demoralization, and Perception of Personal Dignity: An Exploratory Observational Study in a Sample of Patients with a Diagnosis of Breast Cancer

**DOI:** 10.1192/j.eurpsy.2026.10654

**Published:** 2026-07-31

**Authors:** I. Pinucci, A. Maraone, F. Boccardi, M. Pasquini

**Affiliations:** Human Neurosciences, Sapienza University of Rome, Rome, Italy

## Abstract

**Introduction:**

Sleep disturbances are frequently reported among patients diagnosed with breast cancer. Their severity has been associated with depressive symptoms, anxiety, and fatigue. However, to date, no studies have systematically explored the relationship between insomnia and more specific psychological constructs such as demoralization and perceived dignity.

**Objectives:**

This study aimed to explore the psychopathological correlates of sleep disturbances in a sample of patients attending the psycho-oncology outpatient clinic of the Breast Unit at a university hospital.

**Methods:**

Sixty patients with a diagnosis of breast cancer were recruited. Following an initial psychiatric evaluation, the following psychometric instruments were administered: the Insomnia Severity Index (ISI) to assess insomnia severity and impact; the Patient Health Questionnaire-9 (PHQ-9) for depressive symptoms; the Patient Dignity Inventory (PDI) to evaluate different sources of dignity-related distress; and the Demoralization Scale (DS) to assess the presence of demoralization.

**Results:**

The mean PHQ-9 score was 8.31 (SD = 5.30); 27 patients (45%) scored above 9, indicating clinically relevant depressive symptoms. The average PDI score was 39.03 (SD = 14.39). Twenty-one patients (35%) scored above 15 on the ISI, indicative of moderate to severe clinical insomnia. ISI total scores showed significant positive correlations with PHQ-9 scores (r = .478, p < .001), PDI scores (r = .530, p < .001), and all PDI subscales, particularly physical symptom distress (r = .502, p < .001), existential distress (r = .493, p < .001), dependency (r = .573, p < .001), lack of peace of mind (r = .292, p = .024), and lack of perceived social support (r = .343, p = .007). Regarding the Demoralization Scale, ISI scores were significantly correlated with the subscales “loss of meaning” (r = .278, p = .031), “disheartenment” (r = .261, p = .044), and more strongly with “sense of failure” (r = .426, p = .001). The remaining DS subscales (“helplessness” and “dysphoria”) and the DS total scores did not show significant associations with insomnia severity.

**Conclusions:**

In this sample, sleep disturbances emerged as a common clinical issue often associated with depressive symptoms, specific dimensions of demoralization, and dignity-related distress. In our sample, greater insomnia severity was significantly associated with more intense depressive symptoms, increased distress related to dignity in a context of physical symptoms, perceived dependency and lack of social support, and stronger feelings of failure and loss of meaning. These findings support the need for an integrated assessment of sleep and psychological suffering in the care of oncology patients, with the goal of improving overall well-being and quality of life through targeted interventions.

**Disclosure of Interest:**

None Declared

